# Student’s feedback-seeking behavior: A blessing or burden for supervisors

**DOI:** 10.12669/pjms.41.8.11458

**Published:** 2025-08

**Authors:** Irfan Qadir, Rehan Ahmed Khan, Muhammad Khalid, Rahila Yasmeen

**Affiliations:** 1 Dr. Irfan Qadir, MBBS, FCPS, MHPE Department of Medical Education, Riphah Academy of Research and Education (RARE), Riphah International University, Al Mizan Campus, Peshawar Road, Rawalpindi - Pakistan; 2 Dr. Rehan Ahmed Khan, MBBS, FCPS, FRCS, JMHPE, MSc-HPE, MHPE, PhD-HPE Dean Riphah Institute of Assessment (RIA), Professor of Surgery, Riphah Academy of Research and Education (RARE), Riphah International University, Al Mizan Campus, Peshawar Road, Rawalpindi - Pakistan; 3 Dr. Muhammad Khalid, MBBS, FCPS, MHPE Department of Medical Education, Riphah Academy of Research and Education (RARE), Riphah International University, Al Mizan Campus, Peshawar Road, Rawalpindi - Pakistan; 4 Dr. Rahila Yasmeen, BDS, DCPS-HPE, MHPE, PhD-HPE Director Riphah Academy of Research and Medical Education, Professor of Medical Education, Riphah Academy of Research and Education (RARE), Riphah International University, Al Mizan Campus, Peshawar Road, Rawalpindi - Pakistan

**Keywords:** Emotions, Emotion regulation, Feedback seeking behavior, Feedback, Higher education, Supervisor

## Abstract

**Objective:**

Despite the extensively reported benefits of student’s proactive feedback seeking behavior (FSB) from seeker’s perspective, limited attention has been paid to its impact on feedback source i.e supervisors. This study, involving eight supervisors of MHPE program, aimed to explore the impact of student’s FSB on supervisor’s emotions, job satisfaction and emotional regulation strategies used by them.

**Methodology:**

To allow an in-depth exploration of this phenomenon, qualitative phenomenological study design was used, by conducting semi structured interviews with eight supervisors of Masters in Health Professions Education program at Riphah International University, Islamabad between April to June 2024. Thematic analysis was used to create data-driven codes and identify key themes.

**Results:**

The participants expressed that as supervisors, they experienced broad spectrum of positive and negative emotions. However, positive emotions were reported less frequently and were less diverse than negative emotions. All the supervisors indicated the need to regulate their emotions using antecedent-focused and response-focused strategies. Supervisors expressed that FSB enhanced their job satisfaction by virtue of helping their students.

**Conclusion:**

Collectively findings of this study highlight that supervising a Master’s program is a cognitive as well as emotional endeavor. Therefore, faculty development programs should also focus on developing emotional intelligence among supervisors.

## INTRODUCTION

Feedback-seeking behavior (FSB), a term coined by Ashford et al. in 1986, refers to feedback session which is initiated at the request of student himself to determine adequacy of his own performance.^1^ This is contrary to feedback given by teachers/supervisors as part of learning process or an institutional requirement. Over the last 30 years research has shown that FSB is beneficial for feedback seeker.^2^,^3^ such as boosting their performance and creativity, promoting learning and growth as well as building positive images in the organization.^3^,^4^

According to Minnikin et al. it is not well-understood whether supervisors appreciate or resent being sought for feedback.^5^ On the one hand, supervisors may experience positive emotions, as they can gather valuable information aligning with their own work goals. On the other hand, being asked and responding to feedback increases supervisors workload which may elicit negative emotions and require supervisors to regulate their emotions.^5^ This lack of attention to supervisors is surprising given that feedback episodes are dynamic and contain interpersonal interactions between the students and supervisors and will have impact on both sides.^4^,^6^

Despite wide body of literature exploring emotions and feedback from student’s perspective, the impact of student’s feedback seeking on emotional experiences of supervisors and their subsequent work engagement and job satisfaction remains under represented in published literature.^3^,^7^ A comprehensive literature review identified only seven relevant studies: three involving industrial supervisors, two focused on clinical supervisors teaching post-graduate medical trainees, one on PhD supervisors, and one addressing theoretical implications. This gap highlighted the need for more focused research to understand the emotional dynamics of feedback process from supervisor’s perspective. Therefore, in this study, we aimed to explore the impact of student’s proactive feedback seeking on supervisor’s emotions, job satisfaction and emotional regulation strategies used by them.

## METHODOLOGY

Hermeneutic phenomenological study design was used to conduct our study. This methodology facilitated a comprehensive and contextual exploration into supervisors lived experiences of dealing with student’s proactive feedback seeking behavior.^8^ There is no consensus on sample size for qualitative studies. According to literature sample size for phenomenological exploration should be between 5-25 till theoretical saturation of data is achieved.^9^ In accordance with published guidelines for phenomenological studies, purposive sampling was used to recruit eight supervisors of Masters in Health Professions Education Program from Riphah International University, Islamabad, to achieve theoretical data saturation.

### Ethical Approval:

Data collection took place between April to June 2024 after approval from Ethical Review Committee of Riphah International University (Riphah/IRC/24/1023; Dated: February 12, 2024).

Prior to starting data collection systematic review of literature was done to form a semi structured interview guide in accordance with standards set in the AMEE guide 87: developing questionnaire.^10^ Interview guide is presented in Appendix-1. Semi structured interviews were conducted in English language online over zoom and audio recorded. Each interview was between 30 minutes to one hour. In order to gain in-depth understanding, additional inquiries were made and participants were encouraged to elaborate their answers with real life examples. Observations and analytical notes were taken by the researcher during the interview wherever required.

Data was entered as transcribed verbatim of interviews into NVIVO software and then manually coded. To analyze the data, we began by reading the transcripts multiple times, identifying and labeling text segments relevant to the research questions, largely based on participants’ own words. Initial codes were developed and continuously refined through insights from relevant literature^11^ and based on the current data. Working in parallel, we also created case summaries and memos to capture distinctive emotional experiences of each supervisor. Emotions were finally categorized as positive and negative emotions which were broken down into a number of discrete emotions. Various emotion regulation strategies were initially coded and then finally grouped into two broader approaches, i.e., antecedent-focused and response-focused strategies.

Recommendations by Guba et al. were followed to ensure rigor in qualitative research.^12^ To mitigate potential biases, data triangulation was employed with the assistance of co-authors. To mitigate interviewer bias, principal investigator engaged in reflexivity and bracketing to maintain neutrality and used open ended questions to elicit authentic participant responses.^12^ To enhance credibility and trustworthiness of data analysis, the second author coded 50% of the data. The transcribed data was sent back to respondents to check for accuracy.

## RESULTS

Eight faculty members (five females and three males) participated in semi structured interviews. All the participants were medical educationist and were supervisors for Masters in Health Professions Education program. The participants expressed that as supervisors, they experienced variety of positive and negative emotions. However, positive emotions were reported less frequently and were less diverse than negative emotions. All the supervisors expressed when students acted proactively and took charge of their own learning, they felt love, happiness, excitement and were motivated intellectually by students. Sometimes, proactive feedback seeking ignited self-reflection in supervisors. On the other hand, supervisors experience negative emotions when students came unprepared, made silly mistakes despite repeated feedback or did not have insight into their own performance. At instances, poor performance of students would make supervisors question their own competence.

### Table-I: Spectrum of supervisor emotions and representative quotes:

**Table-I:** Spectrum of supervisor emotions and representative quotes.

Emotions		Representative Quotes
Positive emotions	Appreciation	Students are taking responsibility of their own learning. I am always appreciative of it because I am supervising so many students, so it is not possible for me to keep check on everyone. That’s why I always need students who are proactive (P5)
	Happiness & Excitement	Proactive students always make me happy because it is these proactive students who will publish their research and will help you increase your faculty profile (P2)
	Intellectual Pleasure	I like a student pushing me to higher intellectual level and asking questions that force me to think (P4)
	Love	My students are just like my children. I love them like my children. It makes me happy when they approach me for guidance because it’s their project and it’s my project (P4)
	Reflection	Giving feedback makes me think about myself too (P6)
Negative emotions	Anger	When student contacts at inappropriate time like at dinner time and expects me to respond at the same time and then keeps on bugging me until they get an answer, then it makes me angry (P1)
	Defensive	My students are mature learners and would repeatedly request for feedback. But the moment you start giving feedback they will get defensive and you end up explaining yourself that I am just identifying behavior and there is nothing wrong with you but they will not understand (P8)
	Disappointment	What does your feedback mean? (P6)
	Exhausting	Its (feedback) is a very cumbersome thing and has emotional toll on me (P8)
	Frustration	It is frustrating if the student does not have self-awareness or there is issue with student seriousness and lack of management by students (P1)
	Stress	So now when someone asks me for feedback, it makes me stressed and I start anticipating about implications of whatever I would say. So I really need to select and choose my words. It really has an emotional toll on me (P3)
	Question own self	Sometimes it makes you question yourself that I am not capable of giving feedback (P7)

All the supervisors indicated the need to regulate their emotions.

*I almost always have to regulate my emotions because my students are not immature undergraduate students. My students are faculty members and it is sort of a faculty development program **(P5)** Yes, I do regulate my emotions because if I don’t, then it will reflect in their learning. I am their role model and one day they will themselves become supervisors and copy my attitude towards their students **(P4)***

### Table-II: Supervisor emotion regulation strategies with their representative quotes:

**Table-II:** Supervisor emotion regulation strategies with their representative quotes.

Emotion Regulation Strategies		Representative Quotes	
*Antecedent – focused strategies*	Situation modification	Schedule	I am a very busy person and always schedule meetings P4 and P8
		Ground rules	Set clear expectations. This saves both of you a lot of time P6
		Pick Trainees	If I am the program directory then I like to pick students and assign students to supervisors based on personality traits of both supervisors and students P1
	Situation reappraisal	Ault Learners	I do respond to messages from students at odd times. I console myself with fact that my students are adult learners. They are busy clinicians and only get time late at night to work on their thesis and then they message me late…. P3
		Prioritization	If I am in the midst of something and approached for feedback, I will use Eisenhower matrix to decide what is important and what needs my immediate attention… P2
		Empathy	Although I am not happy but I put myself in student’s shoes and try to facilitate my students P7
*Response – focused strategies*	Emotion Suppression	I will not mention my negative emotions unless they have made a big blunder in research otherwise I don’t express my emotions P1	
	Respond later	I do not react to situations. I respond to situations. What I do is that I sometimes write an email to respond but not send it immediately. I keep on reviewing it before finally sending it P2	
	Emotional Intelligence	I am emotionally very intelligent. Supervisorship is about managing people. Supervisors need to have leadership skills. I need to manage my emotions and that of my students too P4	
	Express feelings	I don’t hide my emotions. If I am unhappy, my student will feel it. I am very open about it P5	
	Apathy	Don’t get too attached to students. Be in control of your emotions P7	
	Chunking	If my student has not performed well, I would give him bite sized feedback. So instead of focusing on 200 things, I would tell him to improve on five things first and once they have improved on those things I would tell them to work on next 5 things P2	

Supervisors expressed that proactive feedback seeking enhanced their job satisfaction by virtue of helping their students.

*Students seeking feedback increases self-efficacy and motivation of supervisor **(P1).** It just gives me satisfaction I get to touch so many lives in a positive way (**P8).** I feel accomplished and my self-esteem as a professional is linked to my contribution to student’s work (**P3).***

## DISCUSSION

The main findings of this study highlight that supervision of MHPE students is an emotionally rich experience and variety of emotion regulation strategies are used by supervisors. Emotional response of supervisors predominantly depends on the type of student. Supervisors draw job satisfaction from feedback sessions in terms of being able to help and guide their student.

### Positive emotions:

Positive emotions such as happiness and love emerge when supervisors witness their student’s growth and success. These emotions are often coupled with a deep sense of admiration/appreciation for students who display motivation for self-directed leaning. This finding did not come as a surprise as supervision and teaching share many common domains.^13^,^14^ In contrast to previously reported literature, intellectual pleasure was very frequently reported in our study. We feel that this was due to our students being adult learners who took charge of their own learning. Intense discussions during dissertation preparation would sometimes prompts supervisors to engage in thoughtful reflection and additional research in order to provide effective solutions. Such interactions foster a reciprocal learning experience between supervisors and students, contributing to the sense of intellectual fulfillment experienced by both parties. Similar intellectual pleasure has been was reported by supervisors in humanities.^15^

### Negative emotions:

Concordant with already published research, this study also reported supervisors’ negative emotions more frequently and greater variety compared to positive emotions. Supervisors’ negative emotions such as frustration and exhaustion stem from students’ misunderstandings,^15^ students’ underperformance, lack of intrinsic motivation as well as supervisor’s heavy workloads, and disconnect between supervisor’s expectations and the actual quality of students’ research.^14^,^16^ While some of these challenges were anticipated by our participants, our study also uncovered that supervisors are often particularly frustrated by unexpected breaches of established norms that supervisors had assumed to be followed.^14^ Our study also suggests that negative emotions may pile up if not regulated properly and in a timely fashion. Previous research by Cotterall has shown that discrepancy between supervisors and students working styles can cause stress amongst students.^13^ Our study extends this insight by demonstrating how such mismatches also affect supervisors’ emotional well-being.

### Emotion regulation strategies:

Two broad emotion regulation strategies emerged from our results. Antecedent-focused strategies involve modifying the factors that induce emotions, such as changing the environment or situation. On the other hand, response-focused strategies entail managing emotional reactions and expressions after the emotions have been triggered.^14^ Our participants used both strategies to improve their emotional experiences although distinction between two may be more blurred in practice than it appears in theoretical models. Supervisors would mostly schedule feedback meetings and set ground rules at start of program. Existing body of literature repeated recommends that supervisors empathize with students a finding which is reflected in our results as well. Our participants also employed cognitive change by reappraising situation. They recognized that their students were self-directed adult learners and some of them were faculty members of university too and they deserved to be treated/given feedback in a completely different way.

Similar to results of this study, emotion suppression was the most common response-focused emotion regulation strategy reported by Taxer et al.^14^,^17^ Another important insight from our study is that the participants used emotional intelligence to modulate their emotions by reappraising not only antecedents of emotions, but emotions themselves. Most of the supervisors refrained from venting as they recognized it to be a useless strategy in terms of improving their emotional state. They also displayed high emotional intelligence by recognizing that their own emotional state like feeling frustrated or angry was only natural; hence feeling negative emotions was not reproachable. In terms of practical implications, this study highlights the importance of institutions prioritizing the emotional experiences and mental health of supervisors. In order to support supervisors, institutions should offer in-house counseling services and comprehensive faculty development programs, aimed at enhancing emotional intelligence, helping supervisors manage their emotions, and promoting their overall mental health.^18^

### Limitations:

The limitations of this study include small sample size, the exclusive focus on one discipline (i.e., Health professions education) and the absence of longitudinal data. Another potential limitation is the possibility that we overestimated the emotional intensity of feedback experiences. Our participants were likely more inclined to recount emotionally charged incidents, while less emotional, routine experiences may have been overlooked or forgotten. To address these gaps, future research should focus on examining supervisor’s vis-à-vis supervisee’s emotional experiences captured in situ during actual feedback episode rather than being recall based. It is also worthwhile to explore how the emotions experienced during feedback event impact future relationship between student and supervisor as well as its impact on future feedback events.

## CONCLUSION

The results of this study indicate that supervisors experience broad spectrum of emotions, with negative emotions being more prevalent and varied. The participants utilized both antecedent-focused and response-focused emotion regulation strategies. Collectively these findings highlight that supervising a Master’s program involves not only cognitive skills but also emotional engagement.

Appendix-1: INTERVIEW GUIDE

Thank you very much for making the time to participate in this study. We are going to have a 30 minutes interview, which aims to understand your emotional experience as a MHPE supervisor and your emotional regulation practice. There are no right or wrong answers to these questions. Please answer them based on your own experience.

1) Please briefly introduce yourself.
2) Years of supervising students
3) How frequently you are approached by students asking for feedback on their performance/assignments/tasks ahead?
4) Please describe your emotional experience when student approaches you for feedback on their performance/assignments/tasks ahead?
5) Reflecting on your emotional experiences when asked for feedback, what do you think of your own emotions and the reasons for feeling those emotions?
6) Do you regulate your emotions while providing feedback to students? If so, how? If not, why?
7) How do you provide positive and negative/corrective feedback to students? Do you prefer to remain “mum” rather than deliver corrective feedback?
8) Do you feel stress while providing negative/corrective feedback? If so, why?
9) Does feedback delivery to students, affect your other work engagements? If so how and why?
10) Does providing feedback to students enhance your job satisfaction? If so, how?
11) What are common difficulties you encountered in giving feedback?
12) What advice would you give novice MHPE supervisors regarding the emotional dimension of supervision?
13) Do you have further comments, reflections, and opinions about emotional experiences and emotion regulation of MHPE supervisors?

### Author Contributions:

**IQ and RAK:** Conceptualized the study, developed the proposal and coordinated the project.

**IQ and MK:** Did data collection.

**IQ:** Wrote the original draft and is responsible for accuracy of data and integrity of work

**IQ, RAK and MK:** Analyzed and interpreted the data.

**RAK and RY:** participated in the overall supervision of the project, finalization and revision of the report. All authors have read and approved the final manuscript.
